# Definition and Measurement of Physical and Chemical Restraint in Long-Term Care: A Systematic Review

**DOI:** 10.3390/ijerph18073639

**Published:** 2021-03-31

**Authors:** Lauren M. Robins, Den-Ching A. Lee, J Simon Bell, Velandai Srikanth, Ralph Möhler, Keith D. Hill, Terry P. Haines

**Affiliations:** 1 School of Primary and Allied Health Care & National Centre for Healthy Ageing, Faculty of Medicine, Nursing and Health Sciences, Monash University, Moorooduc Highway, Frankston, VIC 3199, Australia; Angel.Lee@monash.edu (D.-C.A.L.); Terry.Haines@monash.edu (T.P.H.); 2 Centre for Medicine Use and Safety, Faculty of Pharmacy and Pharmaceutical Sciences, Monash University, Royal Parade, Parkville, VIC 3052, Australia; Simon.Bell2@monash.edu; 3 Central Clinical School & National Centre for Healthy Ageing, Peninsula Clinical School, Faculty of Medicine, Nursing and Health Sciences, Monash University, Frankston Hospital, Frankston, VIC 3199, Australia; Velandai.Srikanth@monash.edu; 4 Center for Health and Society, Institute for Health Services Research and Health Economics, Heinrich-Heine-University, 40225 Düsseldorf, Germany; ralph.moehler@uni-duesseldorf.de; 5 Rehabilitation Ageing and Independent Living (RAIL) Research Centre & National Centre for Health Ageing, Faculty of Medicine, Nursing and Health Sciences, Monash University, Moorooduc Highway, Frankston, VIC 3199, Australia; Keith.Hill@monash.edu

**Keywords:** bedrail, belt, mitt, surveillance, lock, gerichair, posey chair, aged care facility, nursing home

## Abstract

This systematic review aimed to identify thematic elements within definitions of physical and chemical restraint, compare explicit and implicit definitions, and synthesize reliability and validity of studies examining physical and/or chemical restraint use in long-term care. Studies were included that measured prevalence of physical and/or chemical restraint use, or evaluated an intervention to reduce restraint use in long-term care. 86 papers were included in this review, all discussed physical restraint use and 20 also discussed chemical restraint use. Seven themes were generated from definitions including: restraint method, setting resident is restrained in, stated intent, resident capacity to remove/control, caveats and exclusions, duration, frequency or number, and consent and resistance. None of the studies reported validity of measurement approaches. Inter-rater reliability was reported in 27 studies examining physical restraint use, and only one study of chemical restraint. Results were compared to an existing consensus definition of physical restraint, which was found to encompass many of the thematic domains found within explicit definitions. However, studies rarely applied measurement approaches that reflected all of the identified themes of definitions. It is necessary for a consensus definition of chemical restraint to be established and for measurement approaches to reflect the elements of definitions.

## 1. Introduction

Frequent use of physical and chemical restraint remains a concern in long-term care facilities internationally [1,2,3,4]. Use of restraint has been justified on the basis of preventing harm to the individual [5,6,7,8] or to others [5,8]. However, adverse consequences of physical restraint include injury, lower cognitive performance, lower performance in activities of daily living (ADLs), higher walking dependence, increased falls, pressure injuries, urinary and faecal incontinence, and death [9,10,11]. Chemical restraint use can lead to decrease in functional and cognitive performance, falls and fractures, excess sedation, and respiratory depression [12,13,14]. Prescribing of antipsychotics has been linked to an increased risk of stroke and mortality [15,16]. Consequently, there have been a range of clinical and policy initiatives over past decades to minimise use of restraint [7,17,18,19,20].

There is considerable variability in rates of physical and chemical restraint between and within countries [21]. Understanding and comparing the impact of different policies and strategies to minimise physical and chemical restraint requires both consistent definitions and measurement. An international consensus definition of ‘physical restraints’ for research was published in 2016. Physical restraint was defined as “any action or procedure that prevents a person’s free body movement to a position of choice and/or normal access to his/her body by the use of any method, attached or adjacent to a person’s body that he/she cannot control or remove easily.” [22]. However, not all research over the past two decades has employed this definition. This may lead to an under or over-estimation of physical restraint prevalence depending on whether the definitions these studies employed had included more or less of the different “restraint” practices as indicated by the international consensus definition of physical restraint. Moreover, it is unclear how well measurement approaches for physical restraints align with both the consensus definition or the definition adopted within the individual studies. Research on chemical restraint is also limited by the absence of a published international consensus definition. Psychotropic medications (e.g., antipsychotics) have approved clinical indications and it is often unclear in prevalence studies what proportion of psychotropic prescribing reflects an intent to restrain [23]. Furthermore, a range of medications have been implicated in restraint, with psychotropic medications including both primary sedatives (benzodiazepines) and medications with sedation as a prominent side-effect (e.g., sedating antidepressants, opioids) [24].

Many studies do not explicitly define physical or chemical restraint. Without clarity on what is being defined as restraint we cannot compare results between studies, we cannot be sure if factors identified as being associated with higher rates of restraint are truly so, and we cannot know if interventions and policies designed to reduce use of restraint actually result in claimed outcomes. Consequently, health professionals will be unable to take actions to reduce use of restraint that are based upon robust evidence, older people will receive care where appropriate monitoring and minimisation of restraint cannot be achieved, and society will struggle to fully appreciate the nature and scale of problems created by use of restraint.

In place of explicit definitions, the measurement approach and reporting of results often form an implicit definition of restraint unique to the individual study. As yet, there has been no work published that has sought to identify and synthesise data from this field in the past two decades relating to the consistency in definitions between studies, consistency between the definitions, measurement approaches and reporting of results within studies, and the reliability and validity of the measurement approaches employed.

The objectives of this study were to (i) identify the different thematic elements within definitions of physical and chemical restraint from studies reporting restraint prevalence or describing interventions to reduce restraint in long-term care facilities, (ii) compare explicit definitions with those that are implicit from the measurement approach used to collect data or approaches for reporting data, and (iii) synthesise the stated reliability and validity of the approaches employed that have been cited in these studies.

## 2. Methods

### 2.1. Design

This was a systematic review with thematic content synthesis of data. The review methods were defined in advance and the protocol was published in PROSPERO (International Prospective Register of Systematic Reviews), ID: CRD42020176726. The systematic review is reported in accordance with Preferred Reporting Items for Systematic Reviews and Meta-Analyses (PRISMA) guidelines [25].

### 2.2. Inclusion and Exclusion Criteria

We sought to identify studies that had measured the prevalence of physical and chemical restraint in long-term care facilities. We also considered definitions of physical and chemical restraint in studies that evaluated interventions designed to reduce restraint use. Studies were included in this review if they (a) published the prevalence of physical, chemical or other forms of restraint use in long-term care facilities (nursing homes, assisted living facilities, residential care, care homes or long-term care wards within these facilities), (b) included a quantitative research design (e.g., randomised controlled trials, clustered randomised controlled trials, quasi-experimental studies, pre and post-test, cohort studies, longitudinal studies, cross-sectional studies or surveys, and (c) included only older adults or reported a participant mean age of at least 65 years.

Studies were excluded if (a) they were published in a language other than English, (b) the restraint use data were reported for any year prior to 2000, (c) the publication was a letter, newspaper article, magazine article, commentary, legal paper, ethics paper, systematic review, case study, a study reporting only qualitative data, poster abstract or dissertation, and (d) the prevalence of psychotropic or other medications were described without establishing an intent to restrain or reduce restraints.

### 2.3. Search Strategy and Selection of Papers

The full holdings of Ovid Medline, PsychINFO, Embase, Emcare, Cochrane Central Register or Controlled Trials, and CINAHL Plus were individually searched up to 21 January 2020. All dates were searched, however, selection of studies was limited to the time frame for reported restraint data rather than publication year. The search strategy included Medical Subject Heading (MeSH) keywords using a modified PICO approach, where ‘Participants’ included terms related to older adults, ‘Intervention’ included terms for physical and/or chemical restraint, ‘Context’ terms included those related to long-term care facilities and ‘Outcome’ terms were related to prevalence (Appendix A). Hand-searching of reference lists of included articles was performed to identify any further studies for inclusion.

Two reviewers (LMR, DCAL) independently screened the title and abstracts of the search results generated. Differences in screening were discussed between the two reviewers until consensus was reached. The full text of all articles that passed the title and abstract screening process were then independently reviewed by the two reviewers (LMR, DCAL).

### 2.4. Data Extraction and Analysis

Investigators (LMR, DCAL) extracted verbatim explicit and implicit definitions of physical and/or chemical restraint presented in the text. Explicit definitions were those preceded by statements indicating that a definition of physical and/or chemical restraint was being presented (i.e., “Physical restraint was defined as…”). Second, the approaches to measurement and presentation of data were identified and extracted. Where an explicit definition was not provided, these data formed an implicit definition for the purpose of this study. Implicit definitions included two categories of information: (a) descriptions of the outcome data collection approach for physical and/or chemical restraint and (b) the reporting of results, both within text and labels in tables used to report results regarding physical and/or chemical restraint use. All definitions reported in the studies were included, regardless of their source or original authorship. All extracted data regarding both explicit and implicit definitions were reported in a spreadsheet for coding. If one reviewer identified text that they felt presented an explicit or implicit definition of physical and/or chemical restraint but the second reviewer disagreed, then a third reviewer (TPH) reviewed the relevant text and made a decision. Information provided by authors regarding the reliability and/or validity of their measurement approach were also extracted.

Thematic content analysis was applied to identify (code) features of explicit and implicit definitions. An inductive, data-driven approach was employed to allow for domains to emerge and subsequent organisation of these into corresponding domains (themes) of physical or chemical restraint [26]. Included papers were re-read, verbatim definitions and descriptions or labels of outcome measures were also re-read and individual elements within each definition were coded. This process was undertaken for each paper by two reviewers (LMR, DCAL) independently. The data extraction tool was then expanded and refined through discussion between the authors throughout the process. The codes were then clustered into thematic areas by three investigators (LMR, DCAL, TPH). Discussions between the three investigators allowed for refinement of the names and descriptions of each thematic area. Bubble plots were used to visually represent the frequency of the presence of each theme along with their conceptualized interconnections, creating a framework of these definitional elements [27]. Each definition could contribute to more than one code and/or theme (see Box 1). A summative approach was used to calculate the total number of definitions that included a particular coded element. The size of each bubble was proportional to the number of definitions that were coded to that code.

Box 1 **An example of how explicit definition data were coded.**

Physical restraint explicit definition: “…are mechanical devices ^1^, materials, or equipments which restrict freedom of movement ^2^ or normal access to one’s body ^3^”. Codes were identified as:

^1^ = “Physical device”

^2^ = “Directly reduce movement”

^3^ = “Restrict normal access to body”

Thematic content analysis was selected as the analysis method over concept analysis as, rather than seeking to define a concept, understand its antecedents, consequences, and related concepts, we sought to understand heterogeneity in domains included in the various definitions and measurement approaches used to investigate the concept. The intent was not to develop a definition or lay claim to what should or should not be included within this definition.

A risk of bias analysis was not conducted for this study as it was not considered relevant to the primary purpose of the systematic review. The authors merely sought to extract and compare the definitions reported within papers and these were considered to be the truth for that paper, therefore no bias was theoretically possible in this regard.

## 3. Results

**Figure 1:**
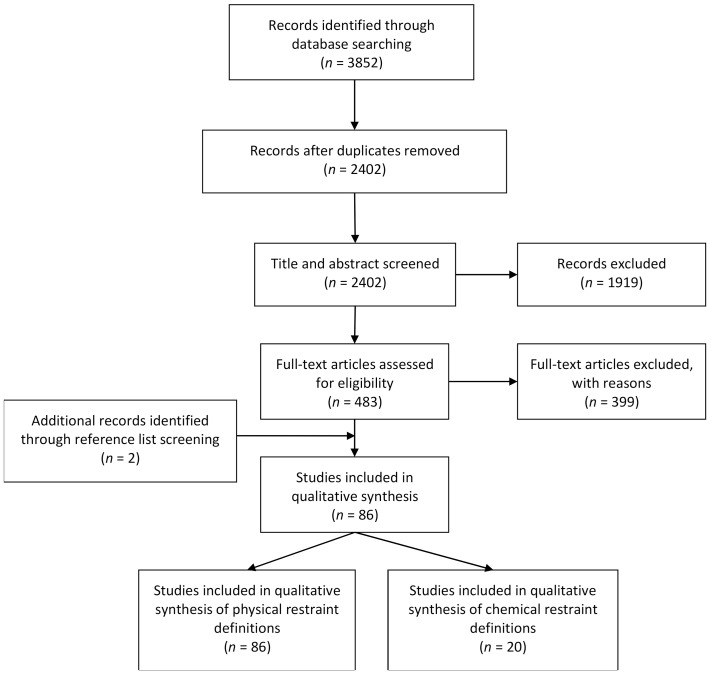
PRISMA flow chart for database search yield.

A PRISMA flow chart commencing with the search yield is presented (Figure 1). A total of 86 papers were included in this review. All 86 papers discussed physical restraint use and 20 of these also discussed chemical restraint use. An explicit definition of physical restraint was provided in 51 papers, while chemical restraint was defined explicitly in four studies. Across all definitions (both explicit and implicit) a total of seven themes were generated following thematic content analysis. The codes from which these themes emerged are presented in the individual bubble plots and an example of how the data was coded is presented in Box 1.

Results were reported first for themes that emerged from studies that used an explicit definition of physical restraint, followed by themes from implicit definitions of physical restraint (identified from their data collection approaches and reporting of results). Results were then presented for chemical restraints in the same order. However, only one bubble plot was included to represent chemical restraint findings (for data collection approach) as there were only limited findings for codes and themes of chemical restraint definitions. Finally, results were reported for the reliability and validity of physical and/or chemical restraint measurement tools as reported in the individual studies where these data were available.

### 3.1. Physical Restraint Explicit Definitions

**Figure 2:**
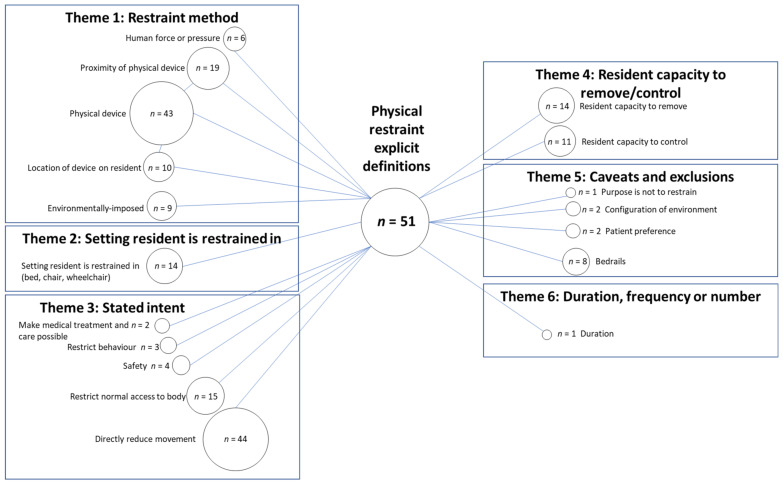
Codes and themes present within explicit definitions of physical restraint. *n* = number of explicit definitions for which this code was identified as being a part of the definition.

The codes and themes identified within reported physical restraint explicit definitions, in a long-term care setting, are presented (Figure 2).

#### 3.1.1. Theme 1: Restraint Method

Explicit definitions of physical restraint frequently included information regarding the method of restraint. This theme included “physical device” which was either a specific device or devices, for example “wrist strap, abdominal belt or ankle brace” [28], or a more general statement, such as “any physical or mechanical device” [5]. Related to this was the code for “proximity of physical device” which described whether the restraints were attached or adjacent to the resident’s body and the “location of device on resident” such as “trunk restraint” and/or “limb restraint”, often with no further detail provided.

The “restraint method” theme also included the application of “human force or pressure” and “environmentally imposed” restraint methods. Examples of “human force or pressure” restraint methods included “the holding of hands, legs or the head during assistance with the activities of daily living…” [29], and “…relational restraint such as force and pressure in hygiene situations…” [30]. Examples of “environmentally imposed” restraint methods included “manipulation of furniture” [31], “being locked in one’s room” [32], locked doors on the ward, such as the ones accessed via keypad [33], or the use of electronic surveillance [7].

#### 3.1.2. Theme 2: Setting Resident Is Restrained in

The setting in which the resident was being restrained was described in 14 explicit physical restraint definitions. This theme included being restrained in bed, a chair or a wheelchair. The setting itself might have provided the restraint, such as “fixation by using deep chairs” [34] or residents may have been held in place in this setting using a specific device, for example “belts tied to a chair” [35].

#### 3.1.3. Theme 3: Stated Intent

The most commonly stated intent of the physical restraint was for the code to “directly reduce movement” of the resident. On some occasions, this code was accompanied by the intent to “restrict normal access to body”, e.g., “deliberately intended to prevent a person’s free body movement to a position of choice and/or a person’s normal access to their body” [36]. Other codes included to “restrict behaviour”, and to “make medical treatment and care possible”. Only four explicit definitions listed reasons reflecting the restraint being used to manage a safety concern.

#### 3.1.4. Theme 4: Resident Capacity to Remove/Control

A number of explicit definitions of physical restraint included the resident’s capacity to remove and/or control the restraint. The level of difficulty experienced in attempting to remove or control the restraint was often described in terms of not being able to remove/control, or not being able to remove/control it easily, e.g., “…cannot control or easily remove” [37]. One definition used the capacity of a resident to remove a restraining device as a criterion for no longer classifying this as use of restraint, “residents can release some restraining devices (e.g., seat belts) and hence, these devices are not considered restraints” [38] and results were reported for those wearing a “potential” restraining device, the percentage of these who were able to release their restraint and an adjusted prevalence rate that excluded those who were able to release it.

#### 3.1.5. Theme 5: Caveats and Exclusions

Caveats, or statements regarding when a device or method was not considered to be a restraint, were included in a number of explicit definitions. Within eight physical restraint explicit definitions it was stated that bedrails were not considered to be a physical restraint. Some studies listed a reason for this, such as “bed rails are often considered safety equipment” [12]. Patient preference was also described as a reason that something would not be considered to be a physical restraint, “The use of bedrails on demand by a resident was not included in this definition” [39]. Two definitions also excluded configuration of environmental factors as being physical restraints “half doors and locked doors forming a barrier or obstacle to keep the older person in a given area, were not considered as physical restraints” [40]. One definition described an exclusion where the device was not being used by the care staff for the purpose of restraint, i.e., “Where restraint devices were used for residents but not for the purposes of restraint, these were not included” [41].

#### 3.1.6. Theme 6: Duration or Frequency

The duration of application of a physical restraint was reported in one explicit definition only. The definition stated that physical restraint was an individual being restrained “by the same mechanism continuously for >2 h...” [2].

### 3.2. Physical Restraint Implicit Definitions Based On Outcome Data Collection Approach

There were 73 reported data collection approaches that allowed domains of implicit definitions, based on outcome data collection approach, to be formed. An example of how an outcome collection approach was coded is presented (in Box 2) for the most commonly applied assessment tool, the Resident Assessment Instrument–Minimum Data Set (RAI-MDS) (25 studies). The wording of the RAI-MDS items used was reported in 12 of the 25 studies. All studies that applied the RAI-MDS were coded using the original survey item wording unless explicitly stated within the study that changes were made to the outcome measure. Four studies reported using data from the Online Survey Certification and Reporting (OSCAR) System, a uniform database of U.S. state nursing home regulatory reviews for facilities completed every 9–15 months [5]. Item F221 is “free from physical restraint” [5]. There were 15 other nursing home quality indicator tools or databases used in different countries to report physical restraint outcome data (e.g., National Nursing Home Survey from the United states, and Aged Care Funding Instrument assessments from Australia). Customised questionnaires developed by researchers were used as the measurement approach in 18 studies. Data was collected by direct observation in 16 studies, while an interview of staff, resident or family was used to collect data in 15 studies. Data extraction from medical records was used in 24 studies. Some studies applied more than one outcome measure approach in order to collect data.

Box 2 **An example of how data collection approach data were coded.**

Section P of the RAI-MDS is labelled ‘Devices and restraints’ and is reported below:

Use the following codes for last 7 days ^1^: 0 = Not used, 1 = Used less than daily, 2 = Used daily ^2^.

Bed rails:

-Full bed rails on all open sides of bed ^3^
-other types of side rails used (e.g., half rail, one side) ^3^
- Trunk restraint ^4^
- Limb restraint ^4^
- Chair prevents rising ^3^

Codes were identified as:

^1^ = “Duration of observation period”

^2^ = “Frequency of use within observation period”

^3^ = “Physical device”

^4^ = “Location of device on resident”

The data collection approach was not reported in enough detail to be coded in 13 studies, e.g., “The use of restraints was determined by the RA at the time of the resident assessment” [42].

**Figure 3:**
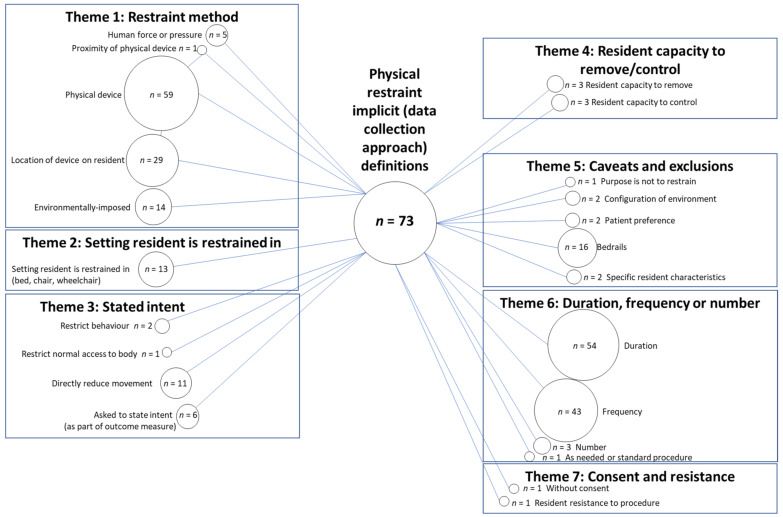
Codes and themes present within implicit definitions, based on data collection approaches of physical restraint. *n* = number of measurement approaches for which this code was identified.

The codes and themes identified within reported data collection approaches, in a long-term care setting, are presented (Figure 3).

#### 3.2.1. Themes 1, 2 and 4: Restraint Method, Setting Resident Is Restrained in and Resident Capacity to Remove/Control

Data collection approaches were considered to reflect the same codes as contained within the themes of ‘restraint method’, ‘setting resident is restrained in’ and ‘resident capacity to remove/control’ as for the explicit physical restraint definitions. Only the number within individual bubbles differed.

#### 3.2.2. Theme 3: Stated Intent

The codes identified within explicit definitions were also found for the theme of ‘stated intent’, with the exclusion of two codes; ‘make medical treatment and care possible’ and ‘safety’. One additional code was created, “asked to state intent”. This included outcome data collection approaches that reported a question about what the intent of the physical restraint was, for example “respondents were asked to summarize the reasons for their use” [35]. Some collection approaches accompanied this with a list of suggestions, e.g., “preventing the resident from falling, wandering, sliding out of their chair, controlling aggressiveness, unknown reason, and also one open ended response alternative.” [32].

One code was created and reported within the theme “Duration or frequency” but was considered to overlap with the ‘stated intent’ theme. This was the code “As needed or standard procedure”. “As needed” was considered to be an intent of the physical restraint.

#### 3.2.3. Theme 5: Caveats and Exclusions

Data collection approaches reported similar caveats and exclusions to explicit definitions with the addition of ‘specific resident characteristics’. These characteristics were listed as a reason for a device not being considered a physical restraint, e.g., “if a resident was indicated as *not mobile in bed* on the restraint-tracking form, the following devices were not considered restraints: a specialty chair with belt, a specialty chair without belt/recliner, or a full lap tray” [43].

#### 3.2.4. Theme 6: Duration, Frequency and Number

In contrast to the one explicit definition that reported ‘duration’, a large number of data collection approaches reported the ‘duration’ of physical restraint data collection period and also ‘frequency’ of use within this period, e.g., “daily use of physical restraints in the previous 7 days” [44].Whether the restraint was ‘as needed or standard procedure’ was also part of the data collection approach in one study, “We also asked whether physical restraints were used for this resident as needed or as a standard procedure” [35].

#### 3.2.5. Theme 7: Consent and Resistance

One additional theme, ‘consent and resistance’ was identified which was not present in the explicit definitions of physical restraint. This was considered to encompass the outcome data collection approach reported in one study. This study used an interview covering 25 items which included “showering or bathing against patient’s verbal or physical resistance” and “feeding a patient against his/her will” [7].

### 3.3. Physical Restraint Implicit Definitions Based On Reporting of Results

**Figure 4:**
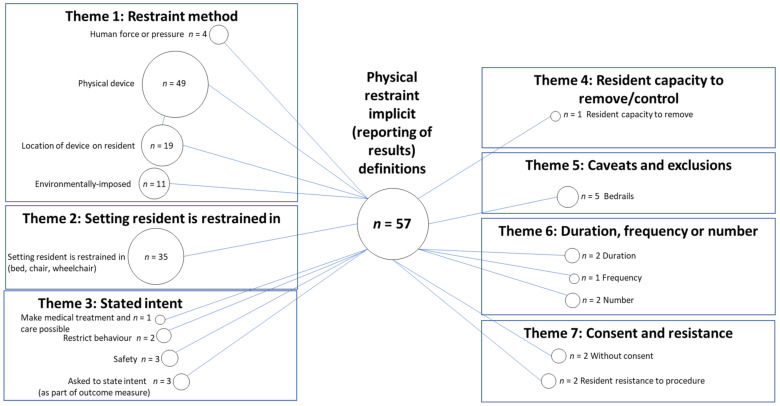
Codes and themes present within implicit definitions, based on reporting of results for physical restraint. *n*= number of reported results for which this code was identified.

There were 57 implicit definitions of physical restraint based on reporting of results that allowed domains of implicit definitions to be formed. The remaining results (29 studies) were reported without any accompanying detail, for example “physical restraints” reported as a percentage of participants who were restrained [45]. The codes and themes identified within reported results of physical restraint, in a long-term care setting, are presented (Figure 4).

#### 3.3.1. Theme 1: Restraint Method

Similar to both explicit definitions, and implicit definitions based on the outcome data collection approach, the restraint method for reporting results included a majority of the same codes. The only code omitted from this theme was for ‘proximity of physical device’, i.e., none of the devices that results were listed for included a description of the measure being on or near the resident.

#### 3.3.2. Theme 2: Setting Resident Is Restrained in

Similar to the explicit definitions, and implicit definitions based on the outcome data collection approach, results were reported in 35 implicit definitions for “the setting the resident was restrained in”. This included in bed, in a chair or in a wheelchair.

#### 3.3.3. Theme 3: Stated Intent

The same codes emerged for implicit definitions based on reporting of results for the theme of ‘stated intent’ for both explicit and implicit definitions based on data collection approach, with the omission of ‘directly reduce movement’. However, fewer studies were coded to each type of ‘stated intent’.

#### 3.3.4. Theme 4: Resident Capacity to Remove/Control

The capacity of a resident to remove their physical restraint was reflected in the results of one study. This study reported results for “liberal” restraints, which they defined as being “corrected for ability to release and includes foot pedals”, whereas the “conservative” results were described as having been “corrected for ability to release and excludes foot pedals” [38].

#### 3.3.5. Theme 5: Caveats and Exclusions

Reported results excluded bedrails as a physical restraint in five studies.

#### 3.3.6. Theme 6: Duration, Frequency or Number

The ‘duration’, ‘frequency’ and ‘number’ of physical restraints were reported across a limited number of studies.

#### 3.3.7. Theme 7: Consent and Resistance

Two studies reported circumstances where consent from the resident was not provided, this included “bedrails without the patient’s consents” [29,46]. Resident resistance was mentioned by another two studies in reported results; “showering or bathing when the resident resists physically” [30], “showering or bathing when the resident resists verbally” [30,46].

### 3.4. Chemical Restraint Explicit Definitions

Only four studies that reported results for chemical restraint contained an explicit definition. Therefore, a limited number of codes were found for chemical restraint explicit definitions. These related to the themes of “restraint method” and “stated intent” of restraint. Other themes that were identified for explicit definitions of physical restraint were either not applicable or not present within definitions of chemical restraint.

#### 3.4.1. Theme 1: Restraint Method

Explicit definitions of chemical restraint reported “medication classes” in three explicit definitions, e.g., “use of psychotropics, hypnotics, or anxiolytics…” [47]. However, no studies listed the specific medications used.

#### 3.4.2. Theme 3: Stated Intent

The intent to ‘control behaviour’ was reported in three explicit chemical restraint definitions. Intent was unclear in two definitions where it was stated that medications had been prescribed ‘without supporting diagnosis’. Inappropriate use was reported in two definitions with one definition mentioning organisational convenience, i.e., “any drug prescribed out of organizational convenience” [23].

### 3.5. Chemical Restraint Implicit Definition Based On Data Collection Approach

There were 16 reported data collection approaches that allowed domains of implicit definitions, based on outcome data collection approach to be formed. One example of how an outcome collection approach was coded is presented (in Box 3) for the Resident Assessment Instrument–Minimum Data Set (RAI-MDS) (approach applied in seven studies). The wording of the RAI-MDS items used was reported in two studies, however, all studies that applied the RAI-MDS were coded using the wording of the original tool unless stated otherwise within the study.

Box 3 **An example of how chemical restraint data collection approach data were coded (extracted from the RAI-MDS).**

Section 0 (Medications), part 4 contains the following information:

Record the number of DAYS ^2^ during last 7 days ^1^; enter “0” if not used.

Note- enter “1” for long-acting meds used less than weekly.

- Antipsychotic ^3^
- Antianxiety
- Antidepressant
- Hypnotic

Codes were identified as:

^1^ = “Duration of observation period”

^2^ = “Frequency of use within observation period”

^3^ = “Medication type/s”

Assessment tools included the RAI-MDS (seven studies) and OSCAR (four studies) as well as the Canadian Institute for Health Information survey (one study), LPZ methodology (in Dutch: Landelijke Prevalentiemeting Zorgproblemen) (one study) and the Flemish Navigator network, a nursing home quality indicator system (one study). A customised questionnaire was applied in three studies, interviews with staff were conducted in three studies, patient records were access for data collection by eight studies and direct observation was carried out in one study.

**Figure 5:**
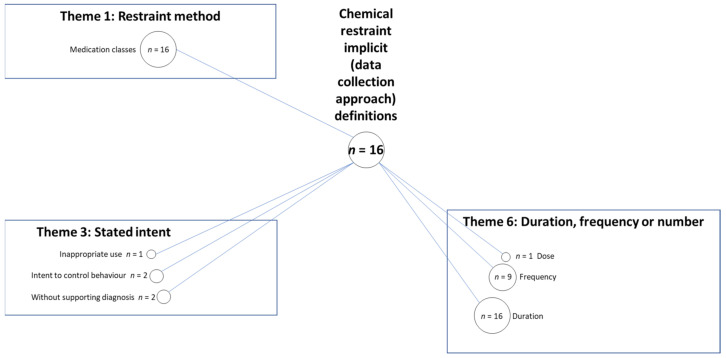
Codes and themes present within implicit definitions, based on data collection approach for chemical restraint.

Three themes emerged from coding of implicit chemical restraint definitions, based on outcome data collection approach. These related to the themes of “restraint method”, “stated intent” and “duration, frequency or number” of restraint (see Figure 5). Other themes that were identified for implicit definitions of physical restraint were either not applicable or not present for chemical restraint.

#### 3.5.1. Theme 1: Restraint Method

The “medication classes” was reported in all 16 chemical restraint outcome data collection approaches. For example, one data collection approach provided “a list… explaining which drugs were considered antipsychotics and benzodiazepines” [48].

#### 3.5.2. Theme 3: Stated Intent

The ‘intent to control behaviour’ was found for two chemical restraint data collection approaches, e.g., “Psychotropic medications are those that affect or alter behaviour” [49]. ‘Inappropriate use’ was reported in one data collection approach, “Inappropriate antipsychotic use was identified” [50]. ‘Without supporting diagnosis’ was reported in two data collection approaches, e.g., “received antipsychotics… without having either MDS-indication of delusions or hallucinations or diagnosis of any psychotic disease (i.e., schizophrenia, mood disorders, or anxiety)” [51].

#### 3.5.3. Theme 6: Duration, Frequency or Number

The ‘duration’ of the observation period for data collection approaches was reported in all 16 studies. ‘Frequency’ of use was referred to in nine data collection approaches, such as was reported in the MDS (see Box 3). ‘As needed or standard procedure’ was reported in four data collection approaches, for example “either a regular neuroleptic drug was prescribed or the resident had consumed the drug as needed…” [45]. The ‘dose’ was included in one data collection approach [23].

### 3.6. Chemical Restraint Implicit Definitions Based On Reporting of Results

There were 16 reported results that allowed domains of implicit definitions to be formed. These related to the themes of “restraint method” and “duration, frequency or number” of restraint. Other themes that were identified for implicit definition of physical restraints were either not applicable or not present for chemical restraint.

#### 3.6.1. Theme 1: Restraint Method

Similar to implicit chemical restraint, based on data collection approach, “medication type/s” was reported in all 16 chemical restraint results. The reported results included the following medication types: antipsychotics, anxiolytic, hypnotic, neuroleptics, antidepressants, benzodiazepines, antiepileptics, psychotropics, and anti-anxiety medications.

#### 3.6.2. Theme 6: Duration, Frequency or Number

One study reported results that included ‘frequency’ data, ‘as needed or standard procedure’ information and ‘dose’ [23].

### 3.7. Reliability and Validity

None of the studies in this review reported validity data for their measurement approach of either physical or chemical restraint use. 46 of the 73 physical restraint studies did not report any reliability of their measurement approach. There were 27 studies that reported data for inter-rater reliability. No studies reported data for intra-rater reliability. Five studies reported inter-rater reliability data derived from the measurement approach used within the individual study (see Table 1), however, three of these studies reported the same reliability data, tested within one study. Eight studies provided a reference to previously published literature and used their reported inter-rater reliability data for their physical restraint measurement approach. The 14 remaining studies that did not provide reliability data for physical restraint measurements, provided reference to previous literature that focussed on a broader suite of nursing home quality indicators rather than restraint use alone.

Only one of the 20 studies that described chemical restraint reported the inter-rater reliability data of their measurement approach (see Table 1). This study provided a reference to previously published literature and used their reported reliability data for their chemical restraint measurement approach.

## 4. Discussion

Definitions of physical restraint have been highly variable in the research describing the prevalence of physical restraint or interventions targeting restraint reduction in long-term care setting. This was comparatively more variable than the definitions of chemical restraint used in the included studies, although substantially fewer studies were identified reporting chemical restraint. Each definition has encompassed various themes relating to aspects of restraint use, with different codes and frequencies of codes within each theme. In particular, the themes that emerged for physical restraint explicit definitions mismatched those for implicit definitions in which “consent and resistance” was only captured in the implicit definitions. In addition, although some studies of physical or chemical restraint reported the reliability of their measurement tools, the majority did not report any reliability or validity data raising the question of potential inaccuracy in their measurement of restraints.

The variable explicit definitions of physical restraint was highlighted by the variety of bubble sizes in Figure 2. It is evident that whether some or all of the themes identified in definitions were used will affect the measurement of restraint. In addition, the specifications for a minimum duration that is required for a resident to be considered to have been restrained, the frequency with which a restraint method is applied and whether caveats apply will also affect the measurement of restraint.

Some of the themes identified in the definitions used in the included studies are missing in the international consensus definition of physical restraints. The consensus definition states that “Physical restraint is defined as any action or procedure that prevents a person’s free body movement to a position of choice and/or normal access to his/her body by the use of any method, attached or adjacent to a person’s body that he/she cannot control or remove easily.” [22] While this definition does encompass the domains of ‘restraint method’, ‘stated intent’, and ‘resident capacity to remove/control’, this definition does not include reference to the ‘setting resident is restrained in’, ‘duration, frequency or number’, ‘consent and resistance’ or any ‘caveats and exclusions’. This will have an impact on measurement and is likely to produce different results to studies using these elements in their definitions.

Study authors generally stated that physical restraint should be defined as including the six themes highlighted in Figure 2, while what was measured generally excluded ‘resident capacity to remove/control’ (with the exception of three studies), and the ‘stated intent’. Many studies were unable to be included in the analysis of reporting of results due to the lack of detail for what results reflected. These results often referred to the physical restraint outcome data collectively as “physical restraint” with an accompanying percentage of participants reported as being restrained. This type of reporting does not inform the reader about what devices were applied or how frequently each device was being applied to residents. Reporting of results that did report some detail generally omitted the themes of ‘stated intent’, ‘duration, frequency or number’, ‘consent and resistance’ and ‘resident capacity to remove/control’. Not only were these findings apparent across this field of study, this mismatch between definitions and data collection approaches and results was also present within individual papers. For example, one study defined physical restraint to be “Any device, material, or equipment attached to or near the residents’ body which cannot be controlled easily or removed by the person and which deliberately prevents or is deliberately intended to prevent free body movement to a position of choice” (encompassing the themes of ‘restraint method’, ‘stated intent’, ‘resident capacity to remove/control’) yet their measurement approach only included “Data on the prevalence of physical restraint use… at 3 time points during 1 day… residents with a physical restraint at 1 or more of the 3 time points were counted as having a restraint” (encompassing the theme of ‘duration, frequency or number’) [60]. This mismatch indicates that the internal face and content validity of measurement approaches within this field can be seriously questioned, as measurement approaches used by researchers do not conform to, or omit, key elements of the definitions of restraint that they themselves have provided. Whether frequency and duration of restraint application are relevant information for a definition of physical restraint is also questionable. The consensus definition does not include frequency or duration elements. It might, however, be useful for outcome measures to reflect intensity of restraint use.

One commonly reported element of explicit definitions of physical restraint is the inability, or relative lack of ease with which a resident can remove or control their physical restraint. However, this element is rarely measured within studies. Only one study directly observed the ability of a sample of residents to remove their restraint as part of the outcome measure, “If a resident had any device that could potentially limit movement, he or she was asked to release this device prior to standing and given graduated prompts to do so”, results were then reported as having been “corrected for ability to release” [38]. While not all of the definitions within this review included the inability of a resident to remove or control the restraint as a defining condition, the consensus review does include reference to this. If future studies are to apply the consensus definition, then this ability should be included as part of the outcome measure for physical restraint.

Most explicit definitions of physical restraint included a statement regarding the intent of the restraint, however, only a limited number took this into account in their measurement approach and few studies reported results for these data. This begs the question of why restraints were being applied in the studies. The consensus definition states that the restraint ‘prevents a person’s free body movement to a position of choice and/or normal access to his/her body’. There are a number of conditions described in some of the reviewed studies that would be considered to be outside this definition. For example, an individual locked in their room might be considered to have ‘free body movement to a position of choice’, however, they may not be free to move to a location of choice as they are unable to leave their room.

The topic of ‘consent’ was not raised in explicit definitions of physical restraint. This topic arose from within outcome data collection approaches and reporting of results, for example, bedrails without the patients consent [29]. This leads to the question of whether the resident consenting to the application of a restraint method disqualifies it as a physical restraint and whether outcome measures and results for physical restraint should reflect this.

A range of outcome data collection approaches were applied across the included studies with only inter-rater reliability having been tested in a limited number of studies to reveal moderate to high reliability. RAI-MDS Version 2 was the most commonly applied outcome measure and does not take into account many of the codes and themes identified in this thematic analysis. Specifically, there is no assessment of environmentally imposed physical restraints, human force or pressure, resident capacity to remove or control the restraint, what the intent of the restraint is or whether resident knowledge or consent has been provided. It has been shown that nursing staff often use less traditional methods of restraint such as removing the resident’s mobility device, keeping the resident inadequately clothed, and even residents feeling so unsafe that they voluntarily lock themselves in their rooms [61]. One study included in this review described how wheelchairs might be classified as a physical restraint if the foot-plates were elevated, “pedals were considered “Elevated” only if they were moved up from a 90° angle perpendicular to the floor” [38]. The RAI-MDS includes a very limited number of the potential methods for physical restraining residents that have been reported in the literature.

In comparison to the physical restraint consensus definition, there is a relative absence of a clear definition of chemical restraint in long-term care. Definitions for chemical restraints included ‘medication classes’, e.g. psychotropics, hypnotics, etc. however, without a consensus definition of which drug classes are considered to be chemical restraint, there is no consistency. Some definitions described the intent of these medications, including to control behaviour or for organisational convenience. Given that these medications often have approved clinical indications then reason for prescription or use is important to consider in defining chemical restraint.

The relative lack of research that clearly identified “chemical restraint” is an important finding given the increasing level of debate about high rates of psychotropic medication use. The intent to ‘control behaviour’ was a theme in the definition of chemical restraint. However, most data sources used in pharmacoepidemiological studies (e.g., prescribing data, pharmacy dispensing data, medication administration charts) do not record the intent of medical practitioners, pharmacists or nurses when prescribing, dispensing or administering psychotropic medications. The term “potentially inappropriate medication use” is often used by researchers when applying explicit or implicit measures of medication appropriateness (e.g., American Geriatrics Society Beers Criteria [62], STOPP/START criteria [63], Medication Appropriateness Index [64]), although potentially inappropriate psychotropic medication use is not necessarily synonymous with chemical restraint. Potentially inappropriate medication use is often defined as use that is associated with more risks than benefits, particularly when safer alternatives exist [65]. This may include use of a medication for an approved indication but at a dose or for a duration that predisposes to adverse drug events. Establishing a clear and measurable definition of chemical restraint will be important moving forward given the recent calls for greater transparency and accountability associated with prescribing, dispensing and administration of psychotropic medications.

### 4.1. Limitations

A limitation of this review was the smaller number of studies located that reported chemical restraint data. One reason for this is that chemical restraint is rarely clearly stated as such. For this study, we limited the inclusion criteria to those studies that referred directly to ‘chemical restraint’ and excluded those for which ‘prevalence of psychotropic or other medications were described without establishing an intent to restrain or reduce restraints’. This may have excluded a number of studies that have investigated chemical restraint but did not refer to it in those terms. Residents may be prescribed psychoactive medications for reasons without restraint, therefore, it is difficult to know if such medications have been prescribed for the purpose of restraint or not unless stated directly. In addition, only studies published in English were included in this review, this may have limited the availability of international studies reporting on physical and or chemical restraint.

### 4.2. Implications

The existing consensus definition of physical restraint was found to encompass many of the domains of physical restraint found within explicit definitions within the literature. However, the measurement approaches applied do not reflect this definition. The consensus definition included domains for a physical device, a stated intent, and resident capacity to remove or control. However, rarely did studies apply measurement approaches that reflected the intent of the restraint, or the capacity of the resident to remove or control it.

It is also necessary to establish a consensus definition of chemical restraint in the long-term care setting. Consistent definitions for both physical restraint and chemical restraint in a long-term care setting will enable the construction of appropriate measurement approaches that are reflective of the elements of individual definitions. Consistent definitions and measurement of restraint would lead to greater potential for uptake of research findings in health care practices. The translation of such research has the potential to reduce and prevent abuse of older adults in long-term care, improve and inform training programs for individuals working with older adults in long-term care and encourages values in-line with person-centred care, thus maintaining the dignity and autonomy of residents and improving quality of life.

## 5. Conclusions

This review identified a range of domains for the construct of physical restraint applied in the literature that largely overlapped with the consensus definition. However, the consensus definition does not discuss several domains we identified, including consent and resistance, or intensity (duration and frequency). The measurement and reporting of restraint appears to be quite distinct from the consensus definition and across the field little is known of the validity and reliability of measures used. Currently no consensus definition for chemical restraint exists. We are largely unaware of reliability and validity of measurement approaches and there has been inconsistency in the definitions applied it is therefore, extremely difficult to be able to monitor the safety and quality of care we provide in long-term care facilities. There is a need for future research to develop measurement approaches that align with accepted definitions of physical restraint given the limited detail or transparency with which previous measures of physical and chemical restraint have been collected and reported.

## Figures and Tables

**Table 1:** Inter-rater reliability data reported in included studies.

Included Studies	Source of Reliability Data		Measurement Approach for Restraint Outcomes	Personnel Conducting Measurement of Restraint Outcomes	Reliability Data Reported
	**Data Derived from within this Study**	**Data Cited from a Previous Study**			
**Studies Reporting Inter-Rater Reliability Data for Physical Restraint Outcome**					
Estevez-Guerra et al. (2017) [1]		√	Direct observation compared to nursing staff report, medical and nursing records	Research assistants and author	Cited Laurin et al. (2004) [52] Kappa = 0.86 (95% CI = 0.73–0.97).
Meyer et al. (2008) [31]		√			
Milke et al. (2008) [43]		√			
Voyer, Verrault, Azizah et al. (2005) [40]		√			
Fitzgerald et al. (2016) [53]	√		Observational measurement tool	Unclear	Kappa = 0.83 (activity during observation period) Kappa = 0.94 (type of restraint used) Kappa = 0.89 (whether patient able to loosen restraint) Kappa = 0.88 (restraint properly applied)
Huizing et al. (2009) [54]	√		Specifically designed physical restraint observation tool	Sample of 2 “trained observers” from the 11 performing data collection	Kappa = 1.0 (observers identified the same restrained residents on one ward)
Huizing et al. (2009) [55]	√				
Gulpers et al. (2011) [56]	√				
Gulpers et al. * (2012) [20]		√			
Kirkevold and Engdal (2004) [29]		√	Specifically designed survey questions	Head of nursing home ward and another carer	Cited Kirkevold, Laake, Engdal (2003) [57] Kappa = 0.5 (physical restraints) Kappa = 0.3 (electronic surveillance and use of force in medical treatment or examination) Kappa = 0.2 (force or pressure to perform ADL)
Mukamel et al. (2008)		√	RAI-MDS	Research nurses and facility nurses	Cited Mor et al. (2003) [58] Kappa = 0.66 (trunk restraint) Kappa = 0.74 (chair that prevents rising)
Pekkarinen et al. (2006)		√			
Schnelle et al. (2004) [38]	√		Wireless thigh monitor for repositioning movements, activity episode for chair and bed. Direct observation in high and low-prevalence restraint homes.	Research staff	Kappa = 0.61 (repositioning movements using wireless thigh monitor) Kappa = 0.82 (activity episodes while in a chair) Kappa = 0.75 (activity episodes while in bed)
					High-prevalence homes: Kappa = 0.64 (in-bed restraints Kappa = 0.65 (chair restraint)
					Low-prevalence homes: Kappa = 0.32 (in-bed restraints) Kappa = 0.65 (chair restraint)
**Studies reporting inter-rater reliability data for chemical restraint outcome**					
Pekkarinen et al. (2006)		√	RAI-MDS	Research nurses and facility nurses	Cited Mor et al. (2003) [58] Kappa = 0.91 (days received antipsychotics)

* Cited Huizing et al. (2006) [59].

## Data Availability

This paper has reviewed data from published papers which are readily available, therefore, we have not made any additional data available.

## Appendix A

**Table A1:** Search Strategy Using a Modified PICO Approach.

P	I	C	O
	“Physical restraint *”		
		“Nursing home *”	
	Restraint *		Prevalence
		“Homes for the aged”	
Aged	“Physical immobili#ation”		Frequenc *
		“Care home *”	
“Older adult *”	“Chemical restraint *”		Quantit *
		Residential facilit *	
Elder*	“Containment measure *”		intensit *
		“Long term care”	
“Older people”	Bedrail *		“Restraint intensity”
		Institutioni#ation	
Geriatric *	Bedchair *		“Restraint status”
		“Assisted living facilit *”	
senior	“Bed alarm *”		“Restraint prevalence”
		“residential care”	
	“Chair alarm *”		“Restraint reduction”
		“residential home *”	
